# High-Dose Aluminum Exposure Further Alerts Immune Phenotype in Aplastic Anemia Patients

**DOI:** 10.1007/s12011-020-02313-6

**Published:** 2020-08-06

**Authors:** Yao Zuo, Xiang Lu, Xiaochao Wang, Suren R. Sooranna, Liju Tao, Shiqiang Chen, Hongwen Li, Dan Huang, Guanye Nai, Hong Chen, Chunfeng Pan, Caihong Huang, Yanmin Pang

**Affiliations:** 1 Department of Hematology, Affiliated Hospital of YouJiang Medical College for Nationalities, Baise, 533000 Guangxi China; 2 Department of Oncology, First People’s Hospital, Nanning, Guangxi China; 3 Department of Surgery and Cancer, Imperial College London, Chelsea and Westminster Hospital, London, SW10 9NH UK

**Keywords:** Aplastic anemia, Aluminum exposure, Immune function, Cytokines

## Abstract

This study explored the relationship between immunological status and clinical characteristics of aplastic anemia (AA) patients to plasma aluminum levels, which were increased after constant exposure to high levels of this metal. Sixty-two AA patients (33 cases with high and 29 cases with low or no exposure to aluminum) and 30 healthy controls were selected for this study. Aluminum in human albumin solution was measured by inductivity coupled plasma mass spectrometry. IL-10, IL-12, IL-17, and INF-γ levels were measured by enzyme-linked immunosorbent assay. The distribution of lymphocyte subsets were determined by flow cytometry. The expression levels of immunoglobulins and complement C3 and C4 were also measured. Exposure to high aluminum raised the levels of serum aluminum in AA patients (*P* < 0.01). The levels of hemoglobin and complement C4 were lower in AA patients with high aluminum exposure (*P* < 0.05 and < 0.01, respectively). The percentage of CD4^+^ T cells and the ratio of CD4^+^/ CD8^+^T cells in peripheral blood in AA patients with high aluminum exposure were higher compared with control AA patients (*P* < 0.05 in both cases), while the percentage of CD8^+^ T cells was significantly lower than that in non-aluminum–exposed AA patients (*P* < 0.05). Compared with non-aluminum–exposed AA patients, the level of IL-10 in the high aluminum–exposed AA group was significantly higher (*P* < 0.05 in both cases). The immunological and clinical characteristics of AA patients from regions of high aluminum exposure are different to those in from non-aluminum areas. These results suggest that high aluminum exposure alters the immune system in patients suffering from AA.

## Introduction

Aluminum is a common metal element in the earth crust, which is widely used in the environment and in our daily lives. Aluminum is a non-essential element of the human body, and long-term accumulation can affect the functions of some human organs [1]. In 1989, the WHO and the United Nations Food and Agriculture Organization (WHO/FAO) officially recognized aluminum as a food contaminant [2]. Subsequently China claimed that aluminum is one of the control indicators of drinking water and that the aluminum content of clean water should be less than 0.2 mg/L [3]. In 1976, Alfrey et al. [4] reported for the first time that aluminum poisoning can cause a nervous system syndrome known as dialysis encephalopathy. In these patients it was found that the content of aluminum in the cerebral cortex and the serum was significantly higher than that seen in normal persons. In recent years, a considerable amount of literature has been published on the amount of harm that aluminum can cause to the human body.

The accumulation of aluminum can cause neurological damage, thus leading to Alzheimer’s disease, brain aging, and neuro-degeneration [5–7]. Chambrun et al. [8] suggested that aluminum may be a risk factor for environmental related inflammatory bowel disease. In addition, Johny Kongerud and Vidar Søyseth [9] found that aluminum exposure was also closely related to respiratory diseases.

Aluminum can also affect the immune system and lead to immuno-toxicity. Long-term aluminum exposure can induce autoimmune diseases. After high or long-term exposure to aluminum, circulating immune complexes are increased, while the red cell immune function is decreased, which is associated with a decrease of the circulating immune complex capacity [10]. Long-term aluminum exposure can also inhibit the immune function of T cells in rats [11]. With respect to humoral immunity, aluminum exposure can affect immunoglobin (Ig) and complement levels in rats [12]. Wei et al. [13] pointed out that aluminum chloride can inhibit the growth density of T cells in vitro, and the higher the concentration of aluminum trichloride, the more significant the inhibitory effect seen. Zhu et al. [14] found that the cellular and humoral immunity of aluminum workers exhibited the tendency of increasing first and then suppressing as aluminum exposure time was prolonged. Surveys conducted by Wang et al. [15] have shown that IgM in serum decreased significantly in the workers of aluminum casting workshops, suggesting that aluminum impacts on humoral immunity. The above studies showed that high aluminum exposure can affect the immune function of people and that this is associated with the aluminum dosage, exposure time, and exposure mode of the person to this metal. However, most of the persons in these studies were healthy individuals. The possibility that high aluminum exposure can affect the immune function of individuals with autoimmune diseases has been rarely studied.

The aluminum content of drinking water in residents exposed to high aluminum is generally higher than that considered to be a hygienic standard, and the serum aluminum levels in high aluminum–exposed area is higher than that seen in low aluminum area [16]. In some outer areas of Baise, a city in the province of Guangxi, China, the content of aluminum in drinking water is more than 300μg/L.

The statistical data gathered from YouJiang Medical University Hospital, which is the main hospital in Baise, over the past 20 years show that the incidence of aplastic anemia (AA) has increased from 296 cases in 1994–2003 to 678 cases in 2004–2014 [17]. As mentioned earlier, aluminum can cause immune toxicity to the human body, but the current information is mainly about the effect of high aluminum levels on the immune function of healthy individuals. In view of the fact that there are more AA cases in the area, and AA is also an abnormal immune mediated hemato-pathological disease, we wondered whether there would be any specific changes in the immune status of AA patients in areas where they are likely to be exposed to high amounts of aluminum. Our previous studies on newly diagnosed AA patients from high aluminum–exposed areas from 2012 to 2013 showed the immunologic manifestation of T lymphocytes was different compared with the AA patients in low aluminum areas [18], and the contents of serum aluminum and lymphocyte subsets showed some correlations.

In order to further confirm this problem and to further explore the immunological characteristics of AA patients in high aluminum–exposed areas and lay the foundation for the diagnosis and treatment of these cases, we collected more AA cases in this study and detected serum aluminum content by using the ICP-MS method. We also determined the peripheral blood lymphocyte subsets using flow cytometry and measured the levels of immunoglobulins and complement C3 and C4 and gathered all relevant clinical data from these patients.

AA is an autoimmune disease characterized by hematopoietic failure. Abnormal proliferation and activation of immune cells and dysregulation of immune-related cytokines can also lead to the occurrence and development of AA [19, 20]. Several studies have revealed that IL-12, IFN-γ, IL-17, IL-10, and several other cytokines play important roles in the occurrence and development of primary AA [21, 22]. In the normal body, studies have shown that high aluminum exposure can affect the levels of TNF-α, IL-22, IL-6, and other cytokines [23]. In this study we also measured several cytokines in patients with AA residing in areas with high exposure to aluminum.

## Materials and Methods

### Research Subjects

Sixty-two cases of AA patients were chosen according to the 2013 version of the AA guidelines [24] from January 2012 to July 2016 in the Department of Hematology, Youjiang Medical University Hospital. Patients with paroxysmal nocturnal hemoglobinuria, myelodysplastic syndrome and acute aplastic crisis, bone marrow fibrosis, and immune-related pancytopenia were not included. None of the patients had other causes of AA. There were 33 men and 28 women, ranging from 15 to 75 years old with a median age of 42.7 years. All the samples taken for immune work were obtained prior to the patients’ treatment with immune suppressive therapy..

The 62 cases of AA patients were divided into 2 groups according to the aluminum concentration of their drinking water and their habitats. There were 33 cases that resided long term in areas of high exposure to aluminum, and the aluminum content of drinking water was more than 300 μg/L [25]. These places are located in aluminum mining areas of Baise, including the towns of TianYang, TianDong, JingXi, and DeBao. All the residents were exposed to aluminum for more than 15 years. These patients were classified as the high aluminum exposure AA group. Twenty-nine cases of AA lived in areas of low aluminum and the concentration of the metal in their drinking water was less than 300 μg/L, and these patients were classed as the low aluminum exposure AA group. These places are in the surrounding areas to Baise and include the towns of Xilin, Tianlin, Lingyun, and Leye. The study also included 30 healthy control healthy individuals living in high aluminum areas and 30 living in low aluminum areas. There was no significant difference in gender and age between AA patients group and healthy control group. All of them joined this study voluntarily with informed consent.

### Experimental Methods

#### Detection of Serum Aluminum by ICP-MS Method

About 3 mL of peripheral blood of the subjects was taken, and the serum aluminum was measured in the Guangxi Analysis and Research Center by the method of ICP-MS.

#### ELISA Detection of Peripheral Blood Cytokines IL-10, IL-12, IL-17, and IFN-γ

About 2 mL of fasting elbow venous blood was withdrawn, and the serum was aliquoted and stored at − 80 °C freezer. IL-10, IL-12, IL-17, and IFN-γ were measured by using ELISA using kits purchased from MultiSciences Biotech Co., Ltd.

#### Detection of Lymphocyte Subsets by Flow Cytometry

FITC-CD3^+^, PE-Cy7-CD4^+^, PE-Cy7-CD8^+^, APC-CD19^+^, and PE-A-CD16^+^CD56^+^ fluorescence labeled mouse anti-human monoclonal antibodies were added to 100 μL fresh heparinized peripheral blood, then mixed and incubated for 15 min. Antibodies were purchased from Becton Dickinson Company. After that, 2 ml of 8.3 g/L NH_4_Cl was added for hemolysis. The samples were then mixed and incubated for 10 min. Then they were centrifuged for 5 min at 1500 rpm. The supernatant was discarded, and 1 mL of PBS was added, and the cells were washed twice following which the number of lymphocytes and lymphocyte subsets was examined by flow cytometric analysis.

### Statistical Methods

The data were analyzed by SPSS version 13.0 statistical software. The Shapiro–Wilk test was used for determining normality. When the data were normally distributed, the results were expressed as the means and standard deviations and one-way ANOVA (Kruskal–Wallis H test) was used for analysis and the *t* test was used for comparisons between two groups. When the data were not normally distributed, the results were expressed as the medians and the Rank sum test was used for analysis. Pearson correlation analysis was used for correlations, and *P* < 0.05 was considered statistically significant.

## Results

### Clinical Characteristics of AA Patients in High Aluminum Exposure Area

The degree of anemia and decrease of bone marrow hematopoietic tissue of AA patients from areas with high aluminum exposure were significantly heavier than in the non-aluminum group. Also, the bone marrow adipose tissue increased more than that in non-aluminum areas. However, there was no difference in the age of onset and gender and the numbers of reticulocytes, leukocytes, neutrophils, platelets, and the degree of bone marrow hyperplasia between two groups (Table 1).

**Table 1:** The clinical characteristics of AA patients in high aluminum–exposed regions

Index	High aluminum exposure AA group	Non-aluminum exposure AA group	*t*	*χ* ^2^	*P*
Age (year)	42.8750 ± 16.89197	42.5000 ± 19.08364	0.081		0.936
Sex	14/19	14/15		0.213	0.644
White blood cells (× 10^9^/L)	2.4656 ± 0.84648	2.5927 ± 0.91803	− 0.567		0.573
Neutrophils (× 10^9^/L)	0.9366 ± 0.50742	1.0963 ± 0.72471	− 1.011		0.316
Hemoglobin (g/L)	49.4688 ± 15.46064	64.8000 ± 18.87235	− 3.509		0.001
Platelet < 10 × 10^9^/L	19 (19/33)	10 (10/29)		3.253	0.071
Reticulocytes	33.9000 ± 32.22288	33.8875 ± 23.18358	0.001		0.999
Bone marrow cytology					
Hypoplastic	27 (27/33)	26 (26/29)		0.752	0.386
Lack of megakaryocyte	26 (26/33)	26 (26/29)		1.326	0.25
Lymphocytosis	24 (24/33)	22 (22/29)		0.078	0.78
Bone marrow biopsy					
Decrease of hematopoietic tissue	28 (28/33)	16 (16/29)		6.491	0.011
Increase of fat tissue	26 (26/33)	15 (15/29)		4.966	0.026

*P* < 0.05 for the difference was considered statistically significant

### Serum Aluminum Levels in AA Patients with High Aluminum Exposure

The median serum levels of high aluminum and non-aluminum exposure AA patients were 767.2 and 257.3 μg/L, respectively, and the median serum levels of high aluminum and non-aluminum exposure control subjects 351.9 and 277.8 μg/L, respectively. The serum level of high aluminum exposure AA patients was higher when compared with non-aluminum exposure AA patients (*P =* 0.006, *a*′ = 0.0083) and the non-exposure control group (*P =* 0.007, *a*′ = 0.0083).

### The Proportion of Lymphocyte Subsets in AA Patients with High Aluminum Exposure

The proportion of CD4^+^ T lymphocytes in the high aluminum exposure AA group was higher than that in the non-aluminum AA group (*P* = 0.026, < 0.05), and CD4^+^/CD8^+^ in the high aluminum exposure AA group were higher than that in the non-aluminum AA group (*P* < 0.0001, *a*′ = 0.0083). The proportion of CD8^+^ T lymphocyte was lower than that in non-aluminum group (*P = 0.03* < 0.05), and the proportion of CD3^+^, CD19^+^, and NK cells in the two groups showed no significant difference. CD4^+^, CD3^+^, and CD8^+^ T lymphocyte ratios in AA patients were significantly higher than the healthy control group (*P* < 0.05), and the proportion of NK cells was lower than that of the healthy group (*P* < 0.01); CD4^+^/CD8^+^ and CD19^+^ lymphocyte ratios in all AA patients and the healthy control group showed no significant difference (Table 2). There was no significant correlations between CD4^+^ and CD8^+^ T lymphocyte populations, CD4^+^/CD8^+^ ratio, and serum aluminum levels (*r* = 0.169, *p* = 0.067; *r* = 0.072, *p* = 0.438; *r* = − 0.039, *p* = 0.679. respectively).

**Table 2:** The distribution of lymphocyte subsets in peripheral blood from AA patients

Index	AA patients	Healthy control group	*t*	*P*
CD4^+^T cells	44.3034 ± 12.12756	39.2946 ± 9.41543	2.518	0.014
CD8^+^T cells	26.8371 ± 10.73067	21.8768 ± 5.53130	3.199	0.002
CD3^+^T cells	73.2096 ± 8.74196	64.8625 ± 10.38917	4.500	0.000
CD19^+^B cells	16.9825 ± 10.35852	18.0821 ± 7.29079	− 0.642	0.523
NK cells	9.9982 ± 6.54969	15.4536 ± 8.66705	− 3.622	0.000
CD4^+^/CD8^+^	1.8000 (median)	1.6500 (median)	− 0.281(Z)	0.779

*P* < 0.05 for the difference was considered statistically significant

### Cytokine Levels in AA Patients with High Aluminum Exposure

**Fig. 1:**
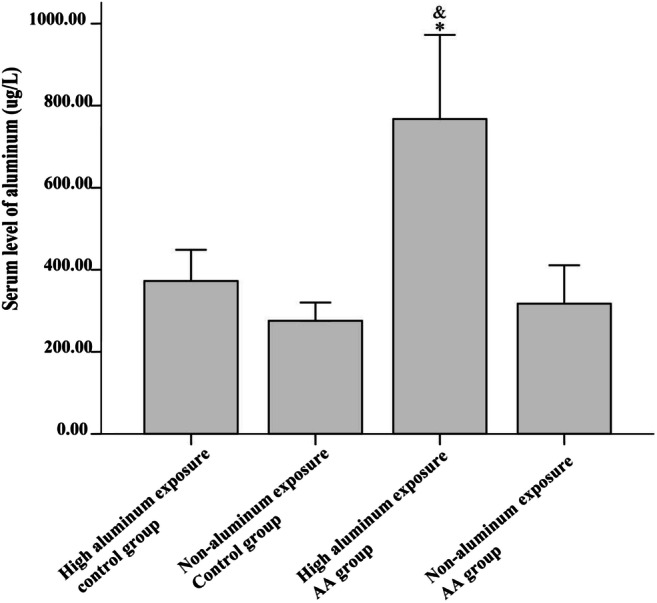
The aluminum levels of AA patients exposed to high amounts of the metal. The serum levels of aluminum of AA patients were measured by ICP-MS method. * represents *P* = 0.006 (*a*′ < 0.0083) when the serum level of high aluminum exposure AA patients compared with non-aluminum exposure AA patients; ^&^ represents *P* = 0.007(*a*′ < 0.0083) when compared with non-exposure control group

**Fig. 2:**
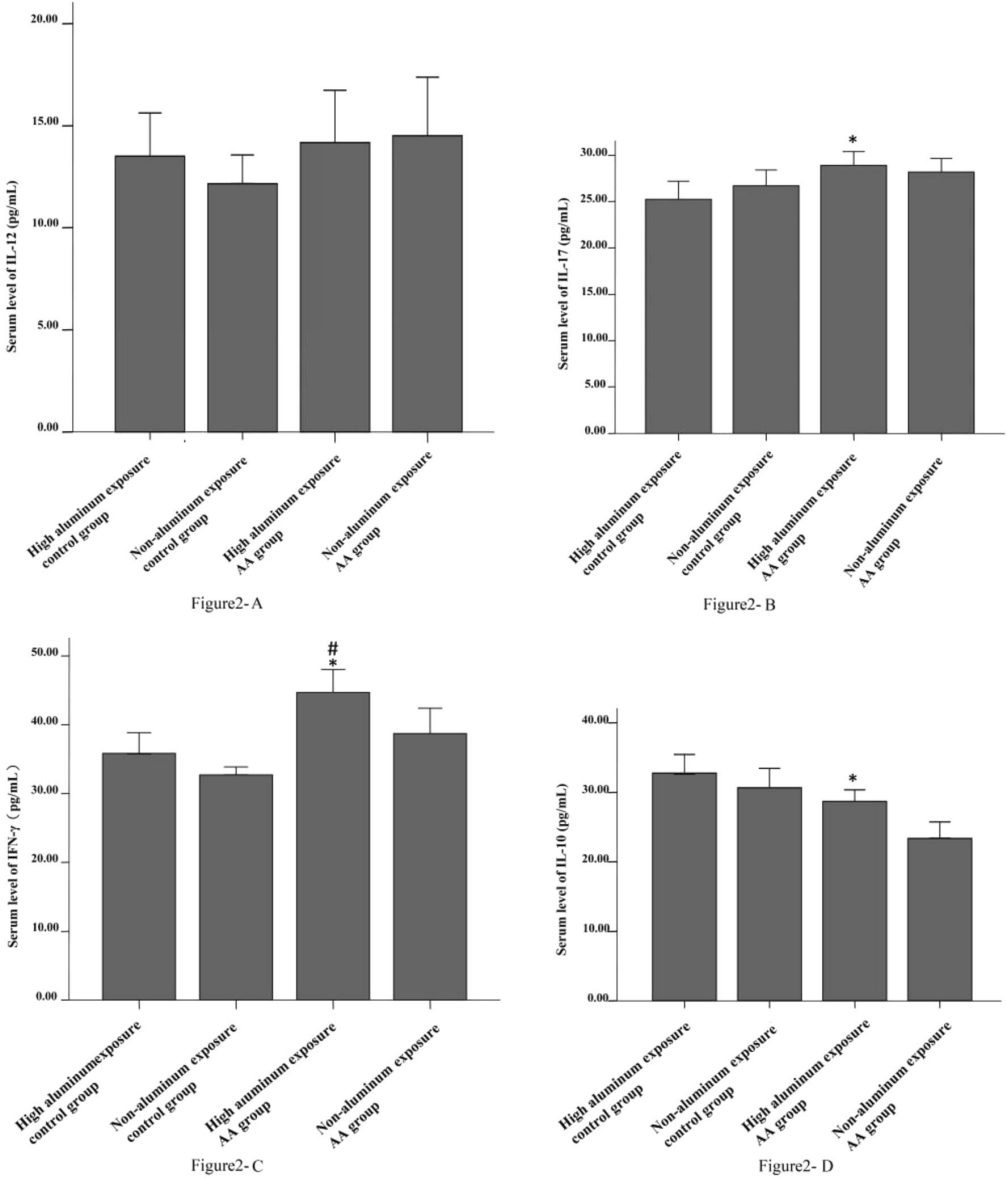
The serum levels of cytokines in high aluminum exposure AA patients. Peripheral blood cytokines, IL-12, IL-17, IFN-γ, and IL-10, were measured by ELISA. **A** IL-12, **B** IL-17, **C** IFN-γ, and **D** IL-10; B* represents *P* < 0.05 when the serum level of IL-17 in high aluminum exposure AA patients compared with high exposure control group. **C** * represents *P* < 0.01 when the serum level of IFN-γ in high aluminum exposure AA patients compared with non-aluminum exposure control group; ^#^ represents *P* < 0.01 when compared with high exposure control group. **D** * represents *P* < 0.01 when the serum level of IL-10 in high aluminum exposure AA patients compared with non-aluminum exposure AA patients

The levels of IL-10 in the high aluminum–exposed AA group were higher than those of non-aluminum AA group. The levels of IL-12 in the high and non-aluminum–exposed AA groups showed no statistically significant difference (Fig. 1). However, in the high aluminum–exposed AA group, IL-17 and IFN-γ were higher than in the high aluminum–exposed healthy control group (both *P* < 0.05), but compared with the non-aluminum–exposed AA group showed no significant difference (Fig. 2A–D). IL-10 levels in AA patients (both high aluminum and non-exposed) were significantly lower than the healthy population (both high and non-aluminum–exposed healthy control groups; *P* < 0.01); IFN-γ and IL-17 levels were significantly higher in the healthy population (*P* < 0.01). There was no difference in the levels of IL-12 between AA patients and healthy controls (Table 3). There was no correlation between serum aluminum levels and cytokine IL-10 (*r* = 0.072, *p* = 0.429).

**Table 3:** The expression of cytokines in AA patients

	Cases	IL-10 (pg/mL)	IL-12 (pg/mL)	IL-17 (pg/mL)	IFN-γ (pg/mL)
AA patients	62	26.12 ± 5.55	14.33 ± 7.32	28.40 ± 2.51	41.84 ± 9.98
Healthy people	60	31.64 ± 7.55	12.87 ± 4.70	26.22 ± 4.32	34.27 ± 6.27
*t*		− 4.61	1.31	3.43	4.99
*P*		0.000	0.190	0.001	0.000

### Immunoglobulin Levels in AA Patients with High Aluminum Exposure

The serum levels of IgE in the high aluminum–exposed AA group showed no significant difference compared with non-aluminum AA group. There was no statistical difference in the levels of IgG, IgA, and IgM among all the groups. The levels of IgE in AA groups were significantly higher than those in the healthy control groups (*P* < 0.01). However, there was no statistical difference in the levels of IgG, IgA, and IgM between these two groups (Fig. 4).

### Complement Levels in AA Patients with High Aluminum Exposure

**Fig. 3:**
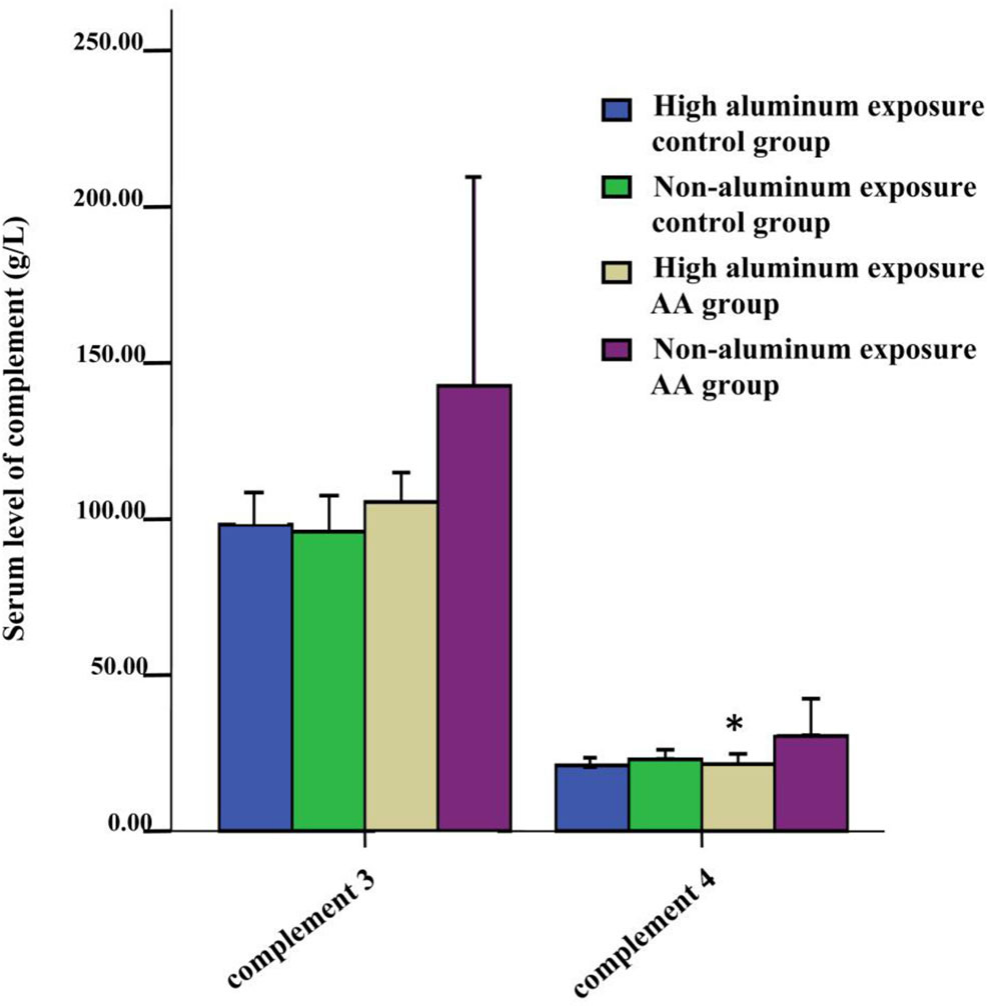
The serum levels of complement C3 and C4 in high aluminum exposure AA patients. * represents *P* < 0.01when the levels of complement C4 in the high aluminum exposure AA group compared with non-aluminum exposure AA group

**Fig. 4:**
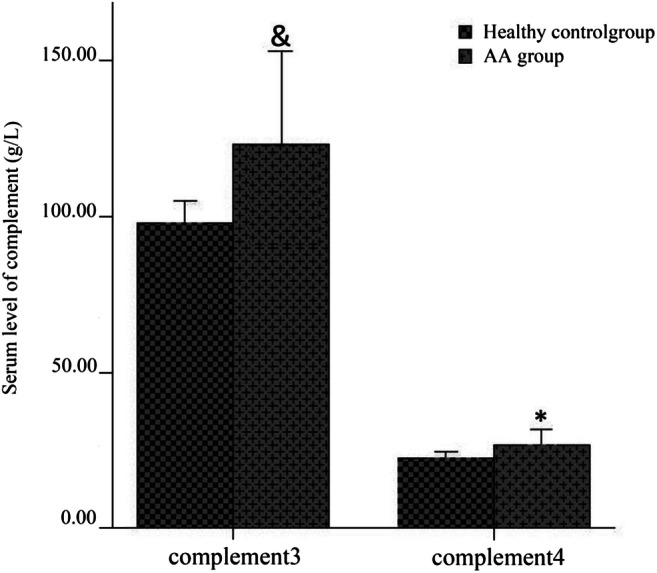
The serum levels of complement C3 and C4 in AA patients. ^&^ represents *P* < 0.05 when the levels of complement C3 in AA patients (high and non-aluminum–exposed groups) compared those in healthy control group (both high and non-aluminum–exposed control groups; * represents *P* < 0.05 when the levels of complement C4 in AA patients (high and non- aluminum–exposed groups) compared with those in healthy control group (both high and non- aluminum–exposed control groups

The levels of complement C4 in the high aluminum–exposed AA group was significantly lower than that in the non-aluminum AA group (*P* = 0.005, *a*′ = 0.0083), while the levels of complement C3 was not significantly different between these two groups (Fig. 3). Complement C3 and C4 levels in AA patients (high and non-aluminum–exposed groups) were significantly higher than those in healthy control group (both high and non-aluminum–exposed control groups; *P* < 0.05) (Fig. 4). There was no significant correlation between serum aluminum levels and complement C4 (*r* = 0.097, *P* = 0.311).

## Discussion

### Serum Aluminum Levels

An epidemiological survey of the Guangxi aluminum mining regions in 2008 showed that the aluminum concentration in drinking water was 408.58 ± 229.27 μg/L and that in non-aluminum mining regions was 239.44 ± 138.51 μg/L [16]. The levels seen in these two areas were significantly higher than that in the general drinking water of China. In this study, the increase in the concentration of aluminum in drinking water in people exposed to high aluminum is probably related to the dramatic changes, which have occurred in the environment of the local aluminum mining areas. Baise City in Guangxi is China’s aluminum industrial base, and it integrates the mining and processing of aluminum mines. The environment in the mining areas has undergone many changes, and it is difficult to protect its groundwater from widespread aluminum pollution. There is also the possibility that the high aluminum concentrations measured in this study includes a small amount of leaching from the blood collection tubes used. Studies have shown that the choice of blood collection tubes can have an influence on the determination of serum trace elements [26]. In order to minimize errors, collection tubes were used uniformly across this study.

### The Incidence and Clinical Characteristics of AA Patients in Areas with High Exposure to Aluminum

AA is a disease due to physical, chemical, and biological or unknown factors that causes damage to bone marrow hematopoietic stem cells. The main clinical manifestations of AA are bleeding, anemia, and infection. Acute AA is characterized by rapid progression, short duration, severe anemia, extensive bleeding, and severe infections, often leading to death. Chronic AA has slow progress and a long course of disease. In AA, the three lineage hematopoietic cells and megakaryocytes are reduced significantly and non-hematopoietic cells including lymphocytes and plasma cells increased.

In this study we found that the Hb levels in AA patients exposed to high aluminum was 49.47 ± 15.46 g/L compared with 64.80 ± 18.87 g/L in AA patients non-exposed to the metal. This was accompanied by a higher degree of anemia in the former AA patients. White blood cells, neutrophils, showed no differences between these two groups of patients which was consistent with our preliminary results [18], and in several other parameters measured. There was a difference in some clinical characteristics between the two groups of AA patients, which may be related to the different immunological characteristics of AA in areas of high aluminum exposure.

### The Percentage of Lymphocyte Subsets in AA Patients in Areas with High Aluminum Exposure

In recent years, the use of anti-lymphocyte or anti-thymocyte globulins (ALG/ATG), cyclosporine A, and other immunosuppressive therapies has been used for patients with AA. These drugs confirmed that this disease is closely related to abnormal immune function [27]. Patients with AA usually have low CD4^+^ T lymphocytes, elevated CD8^+^ T lymphocytes, and an inverted CD4^+^/CD8^+^ ratio. CD4^+^ and CD8^+^ T cell abnormalities are closely related to the development of AA [28, 29], and the proportion of T cell subsets is related to the severity of the condition. CD8^+^ T cells are closely related to hematopoietic failure. After being activated abnormally, they can inhibit colony formation of autologous and allogeneic progenitor cells. The number of activated CD8^+^ T cells in AA patients is increased, and their function is enhanced, and these can have a significant inhibitory effect on the growth of bone marrow cells [30].

A large number of studies have found that natural killer (NK) cells are closely related to the pathogenesis of AA. The proportion of NK cells in the bone marrow and peripheral blood of AA patients was higher [31]. Liu et al. [32] also found that the proportion of peripheral blood NK cells and their subpopulations before treatment in AA patients was lower than those in healthy controls. Sloand et al. [33] found that the decreased activity of NK cells in AA patients may be related to the mutation of the perforin gene. Thus, NK cells may play a role in the immunomodulatory process of AA and lead to the disease. In this study, we also found that CD3^+^ and CD8^+^ T lymphocytes in AA patients were increased and the proportion of NK cells was decreased, suggesting that T cell immune function was hyperactive.

There are several effects of aluminum on T cells. It has been shown that aluminum-containing adjuvant can induce the differentiation of CD4^+^ T cells into Th1/2 to drive inflammation [34], and the differentiation of follicular helper T cells (TFH), which help B cells to participate in humoral immunity and stimulate the body to produce antibodies [35]. Zhu et al. [14] found that the proportion of CD3^+^T, CD4^+^T, and CD8^+^T lymphocyte subsets increased in workers exposed acutely to aluminum, while the expression of T lymphocyte subsets in long-term exposure was inhibited, showing a bi-directional change of characteristics. Wei et al. [13] found that aluminum chloride density had significant inhibitory effects on the growth of human T lymphocytes in vitro and that their growth and aluminum chloride levels was negatively correlated. Zhu et al. [11] also found that the proportion of CD3^+^, CD4^+^ T lymphocytes, and CD4^+^/CD8^+^ in the mice exposed to aluminum was reduced, whereas Gräske et al. [36] believed that aluminum exposure has no effect on T lymphocyte subsets.

Immunoglobulin IgM expression was not significantly different in our study, suggesting that hyper-aluminum AA may be characterized by predominantly immune-dominant T cells, and humoral immunity may not play a significant role. Also, no linear relationship between serum aluminum and CD4^+^, CD8^+^ T lymphocyte ratios, and CD4^+^/CD8^+^ was found.

### Characteristics of Cytokine Expression in Patients with AA in Areas with High Aluminum Exposure

Cytokines play an important role in the pathogenesis of inflammation, tumors, transplant rejection, and autoimmune diseases. Lymphocytes are divided into Th1 cells and Th2 cells according to their different types of cytokines they produce. Th1 cells mainly secrete cytokines such as IL-2, IFN-γ, and TNF-α, and Th2 cells mainly secrete cytokines such as IL-4., IL-5, IL-6, IL-10, and IL-13. Several studies have demonstrated that AA patients express multiple immune-related cytokines [37, 38]. In this study, hematopoiesis appears to regulate the increase of cytokine levels negatively. IFN-γ is a type II interferon and one of the major negative hematopoiesis regulators of AA. Activated T cells and NK cells can produce IFN-γ, which is elevated in patients with AA, and the levels are related to the severity of the disease [39]. Gidvani et al. [40] detected a single-nucleotide polymorphism (SNP) of IFN-γ that may be related closely to the onset of AA. Changes in IFN-γ levels are seen significantly earlier than those in routine blood tests, and it may be used as an indicator for evaluating efficacy and prognosis of AA [41]. In this study, we also found that the levels of IFN-γ expression in AA patients were higher than that in the healthy control group.

Wei X.M et al. [13] reported that aluminum chloride can inhibit the production of IL-2 and TNF-α from cultured human T lymphocytes in vitro. Wang Z et al., [42] reported similar results with chicken T lymphocytes. Subsequent in vivo studies confirmed that serum IL-2 and TNF-α levels were reduced in mice exposed to different doses of aluminum chloride [43] suggesting that aluminum exposure can have an effect on cytokines. The results of this study showed that the IL-10 levels in AA patients exposed to high aluminum was lower than those in the healthy control group, but higher than that in the non-aluminum exposure AA patients. IL-10 is secreted by regulatory B cells and can affect the Th1/Th2 balance [44]. The number of Treg cells in AA patients decreases with low levels of IL-10, and the activation and proliferation of autoreactive T cells are inhibited. IL-10 levels were significantly lower in patients with AA, and elevated in patients with hematopoietic recovery after immunosuppressive therapy [45]. This is similar to our results, and we also found that IL-10 levels in the AA group were higher in areas of high-aluminum exposure, which could have been compensatory increase in these patients.

IL-17 is a pro-inflammatory cytokine that is mainly produced by activated T cells. Tong et al. [46] found that high expression of IL-17 was detected in the peripheral blood and bone marrow of AA patients. Under the stimulation of IL-17, macrophages in patients with AA can secrete more IL-6, IL-8, and TNF-α, which can inhibit bone marrow hematopoiesis and participate in the pathogenesis of AA [47].The serum levels of IL-17 and IL-17 mRNA in patients with AA were significantly higher, and decreased after treatment [48]. In our study, the expression levels of IL-17 in patients with AA were significantly higher than that in the healthy control group.

In summary, this study has explored the characteristics of immune cytokines such as IL-10, IL-12, IL-17, and IFN-γ in AA patients exposed to high aluminum. The expression of IL-10 in patients differed from that seen in primary AA, suggesting that the immune mechanisms in the two groups may be different.

### Immunoglobulin Levels in AA Patients with High Aluminum Exposure

The role of B lymphocytes and its mediated humoral immunity in the development and progression of AA is still not fully understood. Hansen et al. [49] and Gomez-Almaguer et al. [50] have reported cases of successful treatment of AA with rituximab, an immunosuppressive agent primarily directed against CD20-positive B lymphocytes. The serum IgG, IgA, and IgM levels in children with AA were significantly lower than those in controls [51]. In our study, IgE levels were significantly higher in patients with AA than in healthy controls, but the levels of IgG, IgA, and IgM were not significantly different to healthy controls. Workers exposed to short-term aluminum exposure had increased IgG levels, while workers exposed to long-term aluminum had decreased levels, showing a two-way change [14]. Workers exposed to low levels of aluminum had increased IgA levels, while IgM levels and lymphocyte counts were decreased [15]. These inconsistent findings may be related to differences in dose, form, and timing of aluminum exposure. We found no significant differences in the levels of IgG, IgA, IgM, and IgE in AA patients from areas exposed to high or non-aluminum exposure.

### Characteristics of AA Complement System in Areas of High Aluminum Exposure

The complement system has been linked the occurrence and development of various autoimmune diseases. Xu et al. [52] found that the expression of the complement regulatory proteins, CD55 and CD59, were decreased in AA patients significantly compared with controls. Rybakova et al. [53] found that a decrease in complement C3 levels was seen in only a few of the AA cases in his study. In our experiment, we found that the levels of complement C3 and C4 in AA patients were higher than those in healthy controls.

A correlation between aluminum and complement activity was reported previously [54]. Liu et al. [55] reported that the decrease of complement C3 levels and the increase of complement C4 levels were detected in chickens exposed to aluminum chloride. However, Zhu et al. [12] detected a decrease in complement C3 and C4 after the mice were exposed to aluminum chloride. In this study, we first found that the levels of complement C3 and C4 were elevated in AA patients, and the levels of complement C4 was higher in the high-aluminum–exposed AA group compared with AA patients not exposed to this metal. Long-term exposure to aluminum can reduce complement levels. Based on the above findings, it is difficult to conclude the importance of changes complement levels in the presence of aluminum.

### Potential Impact of Aluminum on the Immune System and Anemia

Although our results show that the serum aluminum levels in different groups were higher than those reported previously, the immunological characteristics of each group were found to be different. It is speculated that immuno-toxicity increases with increases in blood aluminum concentrations. Therefore, early detection, identification, and intervention of patients with increased blood aluminum levels or those exposed to high amounts of aluminum will help reduce the risk of aluminum damage to the immune system.

Aluminum can inhibit the activity of ferrous oxidase and binds to transferrin, which affects the utilization of iron. D-amino-levulinic acid dehydratase participates in the synthesis of heme, and aluminum can affect its activity, leading an inhibition of heme synthesis [56]. Aluminum damage to the blood system is common in non-iron deficient anemia and renal anemia. In this study, the number of patients with AA in the high aluminum exposure group was more severe than in the low aluminum exposure group, suggesting that the potential impact of anemia may be greater as blood aluminum levels increase. However, other studies have suggested that serum aluminum is not related to anemia [57] and so it is possible that the aluminum associated defects in heme synthesis also contributed to the higher incidence of AA in the high aluminum areas seen in this study and that the incidence of AA was not truly elevated in these regions but was artificially increased due to the aluminum-induced anemia.

## Conclusions

Aluminum exposure can affect the human immune system. This study found that the clinical characteristics and some immune parameters of AA patients exposed to high amounts of aluminum are different to those not exposed to this metal, suggesting that the pathogenesis of the disease may be different. Whether this phenomenon can be treated as a relatively independent type of AA needs further study.

## Data Availability

The data supporting the findings detailed in this paper are presented in the 3 tables and 4 figures within the main paper, and the full/raw are available from the first author and the corresponding authors.
