# Correction to: High-Dose Aluminum Exposure Further Alerts Immune Phenotype in Aplastic Anemia Patients

**DOI:** 10.1007/s12011-020-02412-4

**Published:** 2020-10-05

**Authors:** Yao Zuo, Xiang Lu, Xiaochao Wang, Suren R. Sooranna, Liju Tao, Shiqiang Chen, Hongwen Li, Dan Huang, Guanye Nai, Hong Chen, Chunfeng Pan, Caihong Huang, Yanmin Pang

**Affiliations:** 1 Department of Hematology, Affiliated Hospital of YouJiangMedical College for Nationalities, Baise, 533000 Guangxi China; 2 Department of Oncology, First People’s Hospital, Nanning, Guangxi China; 3 Department of Surgery and Cancer, Imperial College London, Chelsea and Westminster Hospital, London, SW10 9NH UK

**Correction to: Biological Trace Element Research**

10.1007/s12011-020-02313-6

The article High-Dose Aluminum Exposure Further Alerts Immune Phenotype in Aplastic Anemia Patients, written by Yao Zuo, Xiang Lu, Xiaochao Wang, Suren R. Sooranna, Liju Tao, Shiqiang Chen, Hongwen Li, Dan Huang, Guanye Nai, Hong Chen, Chunfeng Pan, Caihong Huang and Yanmin Pang was originally published electronically on the publisher’s internet portal on 06 August 2020 without open access. With the author(s)’ decision to opt for Open Choice the copyright of the article changed on October 2020 to © The Author(s) 2020 and the article is forthwith distributed under a Creative Commons Attribution 4.0 International License (https://creativecommons.org/licenses/by/4.0/), which permits use, sharing, adaptation, distribution and reproduction in any medium or format, as long as you give appropriate credit to the original author(s) and the source, provide a link to the Creative Commons license, and indicate if changes were made.

